# Early aEEG can predict neurodevelopmental outcomes at 12 to 18 month of age in VLBWI with necrotizing enterocolitis: a cohort study

**DOI:** 10.1186/s12887-021-03056-6

**Published:** 2021-12-20

**Authors:** Si Chen, Xiuman Xiao, Su Lin, Jianghu Zhu, Lidan Liang, Minli Zhu, Zuqin Yang, Shangqin Chen, Zhenlang Lin, Yanli Liu

**Affiliations:** 1 Department of Neonatology, the Second Affiliated Hospital and Yuying Children’s Hospital of Wenzhou Medical University, 109 West Xueyuan Road, Wenzhou, 325027 Zhejiang China; 2 Children’s Rehabilitation Department, the Second Affiliated Hospital and Yuying Children’s Hospital of Wenzhou Medical University, Wenzhou, Zhejiang China

**Keywords:** Preterm infants, Cerebral function monitoring, Necrotizing enterocolitis

## Abstract

**Background:**

Studies have shown that neurological damage is common in necrotizing enterocolitis (NEC) survivors. The purpose of the study was to investigate the predictive value of amplitude-integrated electroencephalogram (aEEG) for neurodevelopmental outcomes in preterm infants with NEC.

**Methods:**

Infants with NEC were selected, and the control group was selected based on 1:1–2 pairing by gestational age. We performed single-channel (P3–P4) aEEG in the two groups. The Burdjalov scores were compared between the two groups. Cranial magnetic resonance imaging (MRI) was performed several months after birth. The neurological outcomes at 12 to 18 months of age were compared with the Gesell Developmental Schedules (GDS). The predictive value of aEEG scores for neurodevelopmental delay was calculated.

**Results:**

There was good consistency between the two groups regarding general conditions. In the 1st aEEG examination, the patients in NEC group had lower Co (1.0 (0.0, 2.0) vs. 2.0 (2.0, 2.0), *P* = 0.001), Cy (1.0 (0.0, 2.0) vs. 3.0 (3.0, 4.0), *P* < 0.001), LB (1.0 (0.0, 2.0) vs. 2.0 (2.0, 2.0), *P* < 0.001), B (1.0 (1.0, 2.0) vs. 3.0 (3.0, 3.5), *P* < 0.001) and T (3.0 (2.0, 8.0) vs. 10.0 (10.0, 11.5), *P* < 0.001), than the control group. Cranial MRI in NEC group revealed a widened interparenchymal space with decreased myelination. The abnormality rate of cranial MRI in the NEC group was higher than that in the control group (*P* = 0.001). The GDS assessment indicated that NEC children had inferior performance and lower mean scores than the control group in the subdomains of gross motor (71 (SD = 6.41) vs. 92 (SD = 11.37), *P* < 0.001), fine motor (67 (SD = 9.34) vs. 96 (SD = 13.69), adaptive behavior (76 (SD = 9.85) vs. 95 (SD = 14.38), *P* = 0.001), language (68 (SD = 12.65) vs. 95 (SD = 11.41), *P* < 0.001), personal-social responses (80 (SD = 15.15) vs. 93(SD = 14.75), *P* = 0.037) and in overall DQ (72 (SD = 8.66) vs. 95 (SD = 11.07), *P* < 0.001). The logistic binary regression analysis revealed that the NEC patients had a significantly greater risk of neurodevelopmental delay than the control group (aOR = 27.00, 95% CI = 2.561–284.696, *P* = 0.006). Confirmed by Spearman’s rank correlation analysis, neurodevelopmental outcomes were significantly predicted by the 1st aEEG Burdjalov score (*r* = 0.603, *P =* 0.001). An abnormal 1st Burdjalov score has predictive value for neurodevelopmental delay with high specificity (84.62%) and positive predictive value (80.00%).

**Conclusions:**

Children with NEC are more likely to develop neurodevelopmental delay. There is high specificity and PPV of early aEEG in predicting neurodevelopmental delay.

## Introduction

Premature infants with neonatal necrotizing enterocolitis (NEC) are at high risk for death or adverse neurodevelopmental outcomes. Understanding the morbidity of NEC with a long-term follow-up would aid neonatologists and pediatric surgeons in making informed decisions in providing care for these patients. However, neurodevelopmental outcomes of premature infants after NEC recovery have not been widely reported, especially in developing countries. Earlier clinical studies were summarized in a systematic review by Ree CM et al. in 2007 [1], which showed that 45% of ELBW infants who had NEC were neurodevelopmentally impaired. Infants with NEC were significantly more likely than infants of similar age and gestation who did not develop NEC to be neurodevelopmentally impaired, including a higher risk of cerebral palsy and visual, cognitive, and psychomotor impairments. Since then, similar findings have been reported in other observational clinical studies [2, 3]. The causes of adverse neurodevelopmental outcomes in premature infants with NEC remain to be determined. It can be multifactorial and linked to perinatal events, severity of disease, surgical treatment and complications and hospitalization.

Amplitude-integrated electroencephalogram (aEEG) is the third generation brain function monitoring technology designed by Maynard in the late 1960s [4] and has been applied in neonatal intensive care units since the 1980s [5]. aEEG involves an amplitude integration of the original EEG, and the output is 6 cm/h, which reflects the background activity. aEEG can also be used in children on ventilation or therapeutic hypothermia. At present, the application of aEEG in the neonatal intensive care unit includes hypoxic ischemic encephalopathy (HIE) and therapeutic hypothermia [6, 7], neonatal convulsion [8], intracranial hemorrhage [9], severe congenital heart disease [10], metabolic diseases [11] and bilirubin encephalopathy [12]. However, the application of aEEG in brain function monitoring of premature infants is still in its infancy. Studies have shown that preterm aEEG background was associated with gestational age and corrected gestational age, and the postnatal brain function of preterm infants showed catch-up development [13, 14]. An early aEEG examination can predict the prognosis of premature neural development [15, 16]. Abnormal aEEG tracings, such as discontinuous low voltage, low voltage, outbreak- inhibition (BS) activities, and lack of sleep-wake cycles, were confirmed to be associated with brain damage based on ultrasound and head magnetic resonance imaging (MRI) in subsequent follow-up. However, most newborns with normal aEEG backgrounds do not have such abnormalities. The purpose of this study was to analyze the relationship of aEEG tracing and neurological prognosis of preterm infants with neonatal NEC.

## Methods

This study was a prospective cohort study. The inclusion criteria were preterm infants of gestational age < 32 weeks who were hospitalized in the level III NICU of the Second Affiliated Hospital of Wenzhou Medical University between October 2017 and October 2018. The control group was set up at a ratio of 1:1 to 2, and the children in the control group were premature infants of the same gestational age admitted at the same time. The exclusion criteria were intrauterine distress, neonatal asphyxia, intracranial hemorrhage (grade III and above), hydrocephalus, periventricular leukomalacia (PVL), HIE, bilirubin encephalopathy, intracranial infection, hypoglycemic encephalopathy, brain malformations, severe bronchopulmonary dysplasia (sBPD), intrauterine growth retardation, complex congenital heart disease, chromosomal abnormalities, and inherited metabolic diseases. All the selected children had their informed consent form signed by their families, and the study passed the hospital ethical review and approval (number LCKY2019-287).

Based on the neonatal postoperative pain assessment scale, the children’s crying, oxygen concentration, vital signs, facial expression, and sleep were assessed, and those who had moderate to severe pain received analgesic treatment. Considering that the t_1/2_ of plasma fentanyl citrate is 3 ~ 4 h, to prevent the effect of analgesics on aEEG, infants who were not given analgesic treatment 12 h before and during aEEG monitoring were included in the experimental group. In infants without NEC, aEEG recordings were performed at the same gestational age and corrected gestational age.

Necrotizing enterocolitis was defined clinically, radiologically or histologically based on modified Bell’s staging criteria [17]. Medical NEC was diagnosed as Bell’s stage II or higher that improved after conservative treatment. Medical management included withdrawal of formula, antibiotic usage and symptomatic support measures. Patients with Bell’s stage IIIB NEC or NEC without bowel perforation who continued to progress after active nonsurgical treatment were treated surgically and classified as surgNEC. In the NEC group, 6-h aEEG recording was conducted during the following four time periods: 48 h (1st), 1 wk. (2nd), 2 wk. (3rd) and 3 wk. (4th) after surgery in the surgNEC group or after diagnosis in the medNEC group. In the control group, aEEG examination was completed synchronously at the same age of correction day.

The aEEG recording (NicoletoneTM Monitor) was conducted by a neonatologist. Based on the international 10–20 system of electrode placement, P3 and P4 were selected as the one-channel electroencephalograms from 2 parietal disc electrodes to obtain aEEG tracings. Only recordings with an impedance < 10 kΩ were analyzed. The frequencies were between 2 ~ 20 Hz and the signal was displayed on a semilogarithmic scale at a speed of 6 cm/h.

The aEEG tracings were reviewed by two neonatologists and an electroencephalographer who had many years of aEEG interpretation and were blinded to the clinical data. Background activity classification was carried out according to the scoring system developed by Burdjalov and colleagues [18], which consists of Co (continuity of the recording), Cy (presence of sleep-wake cycle (SWC)), LB (lower border amplitude score), and B (bandwidth), to objectively assess the developmental maturation of the premature infants to facilitate the assessment of premature infants at different gestational ages. In addition, an aEEG score was determined with regard to the following 3 characteristics: an age-appropriate background pattern according to the reference values in Olischar et al. [19]; the presence or absence of SWC [20]; and the presence or absence of electrographic seizure activity [20]. An aEEG score of ‘1’ or ‘normal aEEG’ corresponded with normality in all 3 categories. A score of ‘2’ or ‘moderately abnormal aEEG’ was obtained when 1 of 3 categories was classified as pathologic and ‘3’ or ‘severely abnormal aEEG’ was defined as an abnormality in 2–3 of the 3 criteria [21].

Each subject underwent a complete cranial MRI 4-5 months after birth. Plain cranial MRI scans were performed by using a head coil in a 1.5 Tesla whole-body imaging system (Philips Gyroscan Intera, Best, the Netherlands.). Based on the findings of MingshuY [22], this study used a semiquantitative scoring system based on MRI morphological findings of brain injury to analyze the cranial MRI images of the included patients, with scores of 3, 2, and 1 for those with moderate/severe, mild, and no scored items, respectively. Among them, ventriculomegaly: the maximum inner diameter of choroid plexus at the level of single/bilateral ventricles 8 ~ 10 mm was considered mild, and > 10 mm was considered moderate/severe; paraventricular leukopenia was counted when there was loss of paraventricular white matter volume; parenchymal abnormalities (NPA) included extracerebral space abnormalities, such as any signal and/or morphological abnormalities in the arachnoid/subarachnoid/epidural space, which were characterized as follows: any abnormality was mild and ≥ 2 abnormalities was considered moderate/severe. Regarding intraventricular hemorrhage, grades I and II were considered mild, and grades III and IV were considered moderate/severe. MRI findings were evaluated by one radiologist who was blinded to the clinical data.

Neurodevelopmental outcomes were assessed using the Gesell Developmental Schedules (GDS) at 12 to 18 months of age. Neurodevelopmental outcomes were classified into 2 groups according to overall developmental quotient (DQ) scores: delay, if DQ < 85 (DQ scores < 70 were defined as retardation; scores of 70–84 indicated moderate delay) and normal, if DQ ≥ 85.

Statistical analyses were performed using IBM SPSS, version 25. Measurement data are expressed as the mean (SD) or median (quartiles). T-tests or Mann–Whitney U tests were used for intergroup comparisons. Categorical data were analyzed with Pearson’s chi-squared test or Fisher’s exact test (when the expected frequency was less than 5). Spearman’s rank correlation analysis was used to analyze the correlations of aEEG to the prognosis of neurodevelopmental function. The risk factors for abnormal neurodevelopmental outcomes were analyzed with logistic binary regression. The receiver operator characteristic (ROC) curve was calculated to evaluate the predictive value of abnormal aEEG scores in predicting neurodevelopmental outcomes and enabled us to calculate the sensitivity, specificity, positive predictive value and negative predictive value. *P* < 0.05 was considered statistically significant.

## Results

A total of 11 preterm infants with NEC were enrolled in NEC group, among which 1 patient in surgNEC died unexpectedly due to pneumonia at 8 months of age when he had been able to turn over. A total of 17 normal preterm patients completed the follow-up study. In the NEC group, there were 10 surgNEC patients. All surgical procedures were uneventful, and vital signs were maintained at normal levels during the perioperative period. All surgNEC patients were exposed to fasting, gastrointestinal decompression (mean: 11.4 days), mechanical ventilation (1 for 16 days and the others for an average of 4.0 days), anti-infective treatment (broad-spectrum antibiotics were used), and correction of anemia (suspended red blood cells were transfused in 8 cases). All cases were transfused with plasma, albumin and intravenous nutritional support and given dopamine therapy. There was 1 medNEC patient, and the medical management included withdrawal of formula for 7 days, intravenous nutritional support, antibiotic usage and symptomatic support. In the control group, the infants were fed normally, and were given intravenous nutrition and symptomatic therapy. The conditions of the two groups in terms of sex, gestational age, birth weight, Apgar score, IVH, highest serum C-reactive protein (CRP), ibuprofen treatment rates in patent ductus arteriosus (PDA), incidence of retinopathy (ROP), BPD, mechanical ventilation (> 96 h), vasoactive drug use, and fasting days are shown in Table 1. As seen in the table, the proportion of males, highest CRP values, mechanical ventilation (> 96 h), vasoactive drug use, and fasting days in NEC group were significantly higher than those in the control group. The highest CRP of the 6 infants in NEC group was greater than 200 mg/L, which was included as 200 mg/L for the statistical analysis. CRP values less than 1 mg/L were included as 1 mg/L for the statistical analysis.

**Table 1:** Epidemiological data of the study group

Clinical characteristics	NEC	No NEC	*P* value
Male	11/11	6/17	0.001^a^
Gestational age, wk	29.07(SD = 1.26)	29.39(SD = 1.15)	0.509^b^
Birth weight, g	1255.45(SD = 177.610)	1323.75(SD = 152.973)	0.274^b^
Small for gestational age	0/10	0/16	/
Apgar-1 min	8.0(7.0, 9.0)	9.00(7.5, 9.0)	0.517^c^
Apgar-5 min	9.0(9.0, 10.0)	10.0(9.0, 10.0)	0.217^c^
IVH grade I or II	3/11	5/17	0.903^a^
IVH grade III or IV	0/10	0/16	/
Highest CRP(mg/l)	141.0(115.0, 200.0)	5.0 (1.0, 11.5)	< 0.001^c^
PDA treated by Ibuprofen	1/11	3/17	1.000^a^
ROP	3/11	2/17	0.353^a^
BPD	3/11	7/17	0.689^a^
Mechanical ventilation, > 96 h	8/11	0/17	< 0.001^a^
Vasoactive drug use	11/11	0/17	< 0.001^a^
Fasting days, d	12.0(9.0, 14.0)	0(0,0)	< 0.001^c^

Values are presented as mean (SD), median (quartiles) or number/total number. ^a^Fisher’s exact test, ^b^T test and ^c^Mann-Whitney U test were used. *P* < 0.05 was considered as statistically significant

The aEEG features of NEC infants included discontinuous background, absence of SWC and abnormal waveforms such as epileptic electrical activity. Based on the scoring system developed by Burdjalov and colleagues [18], the Burdjalov total scores and separate entities, such as continuity, SWC and bandwidth, were compared with the reference indicators (see Table 2). The results showed that the following scores were lower in the NEC group than in the control group: Co (1.0 (0.0, 2.0) vs. 2.0 (2.0, 2.0), *P* = 0.001), Cy (1.0 (0.0, 2.0) vs. 3.0 (3.0, 4.0), *P* < 0.001), LB (1.0 (0.0, 2.0) vs. 2.0 (2.0, 2.0), *P* < 0.001), B (1.0 (1.0, 2.0) vs. 3.0 (3.0, 3.5), *P* < 0.001) and T (3.0 (2.0, 8.0) vs. 10.0 (10.0, 11.5), *P* < 0.001). In the 2nd aEEG, Cy (3.0 (2.0, 3.0) vs. 4.0 (3.5, 5.0), *P* = 0.002), B (3.0 (2.0, 3.0) vs. 3.0 (3.0, 4.0), *P* = 0.009) and T (10.0 (8.0, 10.0) vs. 11.0 (10.5, 13.0), *P* = 0.002) were significantly lower in the NEC group than in the control group. In the 3rd aEEG, Cy (3.0 (3.0, 4.0) vs. 5.0 (4.0, 5.0), *P* = 0.009) and T (11.0 (10.0, 12.0) vs. 13.0 (11.5, 13.0), *P* = 0.013) were significantly lower in the NEC group than in the control group. In the 4th aEEG recording, there were no significant differences between the two groups across indices.

**Table 2:**
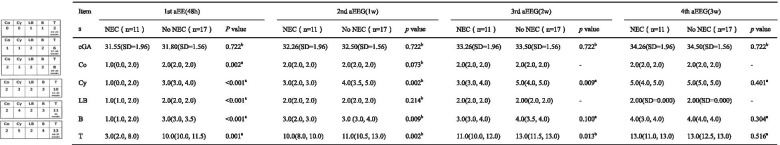
Comparison in aEEG scoring system in two groups

Background activity classification of aEEG was carried out according to the scoring system developed by Burdjalov and colleagues(see the left table). aEEG was conducted 48 h (1st aEEG), 1wk (2nd aEEG), 2wk (3rd aEEG) and 3wk (4th aEEG) after surgery in surgNEC or after diagnosis in medNEC. In the control group, aEEG examination was completed synchronously at the same age of correction day. cGA, corrected gestational age. Co, continuity of the recording (0-2); Cy, presence of cycling (0-5); LB, lower border amplitude score (0-2); B, bandwidth (0-4); T, total score (0-13). ^a^Mann-Whitney U test was used. ^b^T-test was used. *P* < 0.05 was considered statistically significant

Cranial MRI was performed in two groups in 4–5 months old. A total of 11 infants in NEC group, 17 infants in control group had undergone cranial MRI examination. There were no significant abnormalities in the control group. The rate of abnormalities on cranial MRI was higher in the NEC group than in the control group (*P* = 0.001). The magnetic resonance score was significantly higher in NEC group than in control group (2.0(1.0, 3.0) vs. 1.0(1.0, 1.0), *P* = 0.001). Abnormal cranial MRI findings in the NEC group included abnormal extracranial space (5/11), delayed myelin development (2/11) and intracranial hemorrhage (1/11).

The GDS DQ assessment indicated that NEC children had inferior performance and lower mean scores than the control children in the subdomains of gross motor (71 (SD = 6.41) vs. 92 (SD = 11.37), *P* < 0.001), fine motor (67 (SD = 9.34) vs. 96 (SD = 13.69), *P* < 0.001), adaptive behavior (76 (SD = 9.85) vs. 95 (SD = 14.38), language (68 (SD = 12.65) vs. 95 (SD = 11.41), *P* < 0.001), personal-social responses (80 (SD = 15.15) vs. 93 (SD = 14.748), *P* = 0.037) and overall DQ (72 (SD = 8.657) vs. 95 (SD = 11.073), *P* < 0.001). Regarding DQ in the NEC group, there were 2 children with scores < 70, 7 children with scores 70–84, and 1 child with a normal score (=85). In the control group, there were 4 children with scores of 70–84 and 13 children with normal scores (≥85). Logistic binary regression analysis was performed for the influencing factors of poor neurodevelopmental outcomes. The factors included in the study were financial difficulties, parental education (below middle school), maternal age ≥ 35 years, male sex, hCRP, platelets < 100 × 10^9/l, and NEC. After univariate analysis, statistically significant factors were included in the logistic binary regression analysis, and the results revealed that NEC patients had a significantly increased risk of abnormal neurodevelopmental outcomes compared to normal neurodevelopmental patients (aOR = 27.00, 95% CI = 2.561–284.696, *P* = 0.006) after adjusting for male sex, hCRP> 50 mg/l and platelets < 100 × 10^9/l.

Based on Spearman’s rank correlation analysis, there was a positive correlation between the 1st Burdjalov scores and neurodevelopmental outcomes (*r* = 0.476, *P =* 0.012) but no significant associations with the 2nd Burdjalov scores (*r =* 0.251, *P* = 0.207) or 3rd Burdjalov score (*r* = 0.211, *P* = 0.249). The 4th Burdjalov scores all reached the normal level relative to cGA in both groups. aEEG scores were classified as 1 (normal) and 2 (abnormal). When we worked on the assumption that infants with abnormal 1st aEEG scores to postconceptional age would be likely to suffer from neurodevelopmental delay and infants with normal aEEG scores would be neurologically normal later in life, the area under the receiver operator characteristic curve (ROC) was 0.806 (*P* = 0.010), the sensitivity was 61.74%, the specificity was 84.62%, the positive predictive value was 80.00%, and the negative predictive value was 68.75%. The correlations between aEEG scores and magnetic resonance scores were evaluated by Spearman’s rank correlation analysis, and it was concluded that the first Burdjalov score was negatively correlated with magnetic resonance scores (*r* = − 0.397, *P* = 0.040).

## Discussion

In this study, we investigated the clinical characteristics, aEEG findings, MRI images, and neurological assessment of premature infants with NEC at 12 to 18 months of age. Finally, the risk of abnormal neurodevelopmental outcomes in infants with NEC and the predictive value of aEEG for neurodevelopmental delay were analyzed.

As an acute phase protein that increases during the inflammatory process, serum CRP concentrations are commonly used as a surrogate marker for infection. NEC can cause a severe overwhelming systemic inflammatory response. Several clinical studies have shown associations between infection and cerebral injury [23–28], and that the inflammatory response generated in response to NEC can result in clinical decompensation with brain injury or death [29]. In this study, Subjects were included based on strict inclusion criteria. The infants enrolled in the two groups were preterm infants of appropriate gestational age whose GA < 32 wk. Patients were excluded for related conditions that could affect brain development. At the same time, we compared the two groups of children in terms of gestational age, birth weight, Apgar score, incidence of IVH, ROP and BPD, and there were no significant differences. In our study, the infants in NEC group showed a significant increase in CRP levels after NEC and even went through septic shock. Their mechanical ventilation time, vasoactive drug use rate and fasting days were significantly higher than those of the control group. The highest CRP level in the control group was significantly lower than that in the NEC group. Severe infection is the characteristic of infants in NEC group, then how about the brain injury?

We chose aEEG as an examination metrod for early assessment. Studies have shown that aEEG scores of premature infants with brain injury were lower than those at the corresponding gestational age or adjusted gestational age [30, 31]. Recent studies have pointed out a correlation of early aEEG parameters with short-term and long-term neurological outcomes [15, 21, 32]. Cerebral maturation measured by Burdjalov scores and onset of cyclicity during the early NICU course were associated with cerebral injury and other perinatal exposures [33]. Early aEEG with moderate/severe abnormalities in the background was associated with severe structural lesions detected in imaging examination in very low birth weight infants [34]. In aEEG tracings, discontinuous background or BS patterns are the results of brain damage. The presence of SWC in preterm babies matures with age, which has been shown by multiple studies [35] and is another important parameter for evaluating cerebral function. To assess the impact of NEC on preterm infants’ brain development at an early stage, we conducted aEEG examination at different postoperative time periods without using sedative or pain relief drugs. Based on the study by Lisanne J. S., recovery to preoperative brain activity occurred within 24 h after cessation of anesthesia in most preterm infants [36]. In our study, the first examination was scheduled 48 h after surgery or diagnosis, and rechecked at 1 wk., 2 wk. and 3 wk. after surgery. Most infants with NEC showed abnormalities such as voltage discontinuity, absence of SWC, bandwidth enlargement or electrographic epilepticus, and the total scores of the first aEEG and the median of each item lagged behind the scores from infants at the same gestational age by more than 2 weeks, and the differences between the two groups were statistically significant. There were also significant decreases in the total scores of the second and third aEEG in the NEC group, but the median score had reached the normal value of the corresponding gestational age, indicating that the catch-up phenomenon of brain function disappeared in the NEC group, but this was not indicative of abnormalities. Therefore, this significant difference has no clinical value. In addition, we discovered that one NEC patient with intracranial hemorrhage developed seizures. Unfavorable outcomes following seizures in preterm infants include death, epilepsy and neurodevelopmental impairment [37].

Neurodevelopmental outcomes are of importance in preterm NEC infants, but after discharge, these outcomes may be influenced by many factors, such as nutrition, socioeconomic status, parents’ education level and early intervention [38–40]. Indeed, some children are even unable to live with their parents. All these factors may affect children’s cognitive and social abilities. While cranial MRI examination at term-equivalent age can avoid these factors and show brain development at the structural level. At the same time, we compared the two groups of children in terms of economic factors, parental education, maternal age, and family income, and there were no significant differences. Neonatal brain development is in a period of glial cell maturation and white matter fiber myelin formation, during which brain biochemical composition and water content change greatly, with complex MRI signal changes. Brain MRI in preterm infants is characterized by thin cortex, high water content in white matter (decreased myelination) and widened interparenchymal space. It has been shown that preterm infants with surgNEC showed severe WMI on brain MRI [41]. And in our study, 6/11 NEC infants, including one medNEC patient, had brain MRI images with some of the above anomalies, which might demonstrate neurodevelopmental delay.

Then how about the neurodevelopmental outcomes of these premature infants? In this study, neurodevelopmental outcomes were assessed at 12 to 18 months of age using GDS in the NEC group. It has been shown that the mental and motor development of NEC survivors has been shown to be significantly lower than that of normal premature infants [42–45]. A follow-up study at age 7 found that children with NEC had an increased incidence of cerebral palsy and hyperactivity, as well as impaired reading, writing, math and social skills [46]. Our findings are consistent with the above results. The DQ scores for gross motor, fine motor, adaptive behavior and language were significantly lower in the NEC group than in the control group, indicating the existence of neurodevelopmental delay. The NEC patients had a significantly increased risk of abnormal neurodevelopmental outcomes compared to non-NEC patients after adjusting for sex, hCRP and platelets numbers. Regarding sex, based on coincidence, all infants in the NEC group were male. Does NEC have gender susceptibility? Not currently supported [47].

Can aEEG be used to predict neurological outcome? Our study indicated that neurodevelopmental delay was associated with 48 h aEEG Burdjalov scores. We found a high specificity of 84.62% and a positive predictive value of 80.00% for abnormal early aEEG (48 h) with respect to an adverse neurological prognosis. In addition, there was a negative correlation between the magnetic resonance and the 1st aEEG scores; that is, the higher the magnetic resonance score was, the lower the aEEG score was. This showed that aEEG examination in the early stage of brain injury has certain predictive value for brain imaging abnormalities as well as prognosis, which is worthy of further study.

The shortcoming of this study is that the sample size was relatively small. This is because of the gradual improvement in premature infant management in Wenzhou area and of the remarkable achievements in the prevention and treatment of complications. In addition, NEC infants were not divided into the surgery group and the medicine group, and we suggest further studies in qualified units.

## Conclusions

In summary, early aEEG 48 h after NEC diagnosis or surgery suggested a discontinuous background, absence of SWC and abnormal waveforms, such as epileptic electrical activity. Cranial MRI can reveal neurodevelopmental problems or severe intracranial hemorrhage. Prognosis at 12 to 18 months of age suggested that NEC children are more likely to develop neurodevelopmental delay. There was high specificity and PPV of early abnormal Burdjalov scores (48 h) in predicting neurodevelopmental delay.

## Data Availability

The datasets generated and analysed during the current study are not publicly available but are available from the corresponding author on reasonable request.

## Abbreviations

aEEG: Amplitude-integrated electroencephalography
NEC: Neonatal necrotizing enterocolitis
GDS: Gesell Developmental Schedules
DQ: Development quotient
BPD: Bronchopulmonary dysplasia
CRP: C-reactive protein
MRI: Magnetic resonance imaging
